# Allyl isothiocyanate dry powder inhaler based on cyclodextrin-metal organic frameworks for pulmonary delivery

**DOI:** 10.1016/j.isci.2022.105910

**Published:** 2022-12-30

**Authors:** Nianxia Sun, Min Zhang, Wentao Zhu, Pingping Song, Tingting Dai, Peng Huang, Zhili Han, Dianlei Wang

**Affiliations:** 1 School of Pharmacy, Anhui University of Chinese Medicine, Hefei, Anhui 230012, China; 2 Anhui Province Key Laboratory of Pharmaceutical Preparation Technology and Applicaiton, Hefei, Anhui 230012, China; 3 Anhui Province Key Laboratory of Research & Development of Chinese Medicine, Hefei, Anhui 230012, China; 4 Anhui Province Key Laboratory of Chinese Medicinal Formula, Hefei, Anhui 230012, China

**Keywords:** Chemistry, Drug delivery system, Pharmaceutical science, Supramolecular chemistry

## Abstract

In this study, allyl isothiocyanate (AITC) was prepared as the dry powder inhalation by loading cyclodextrin metal-organic framework (CD-MOF) to enhance pulmonary delivery. β-CD-MOF and γ-CD-MOF both could be used to carry AITC with the optimal loading conditions (50˚C, *n*_CD_: *n*_AITC_ = 1:7, 7 h). Compared with β-CD-MOF, γ-CD-MOF had more advantages in AITC loading due to its high drug loading and stable crystal morphology. The particle size and the mass median aerodynamic diameter of γ-CD-MOF-AITC were accorded with the aerodynamic characteristics of lung inhalation. γ-CD-MOF-AITC might be deposited effectively in the deep lung, and the release rate of AITC reached over 90% within 5 min. Meanwhile, it had good pulmonary local tolerance, permeability, and no significant toxicity. Such results indicated that γ-CD-MOF could be used as a dry powder inhaler carrier to deliver safely AITC to lung and increase its pulmonary absorption.

## Highlights

• AITC was prepared as dry powder inhalation by loading CD-MOF
• Compared with β-CD-MOF, γ-CD-MOF had more advantages in AITC loading
• γ-CD-MOF could safely deliver AITC to lung and increase its pulmonary absorption

## Graphical abstract

**Figure:**
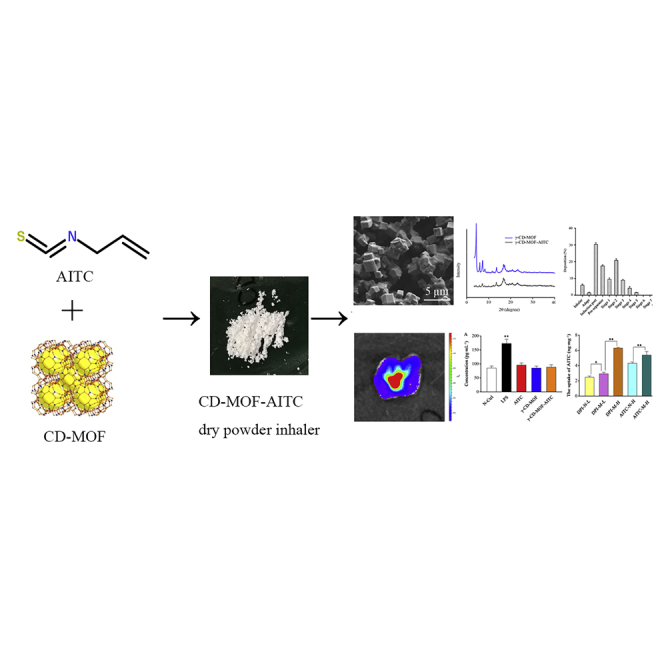

## Introduction

**Scheme 1:**
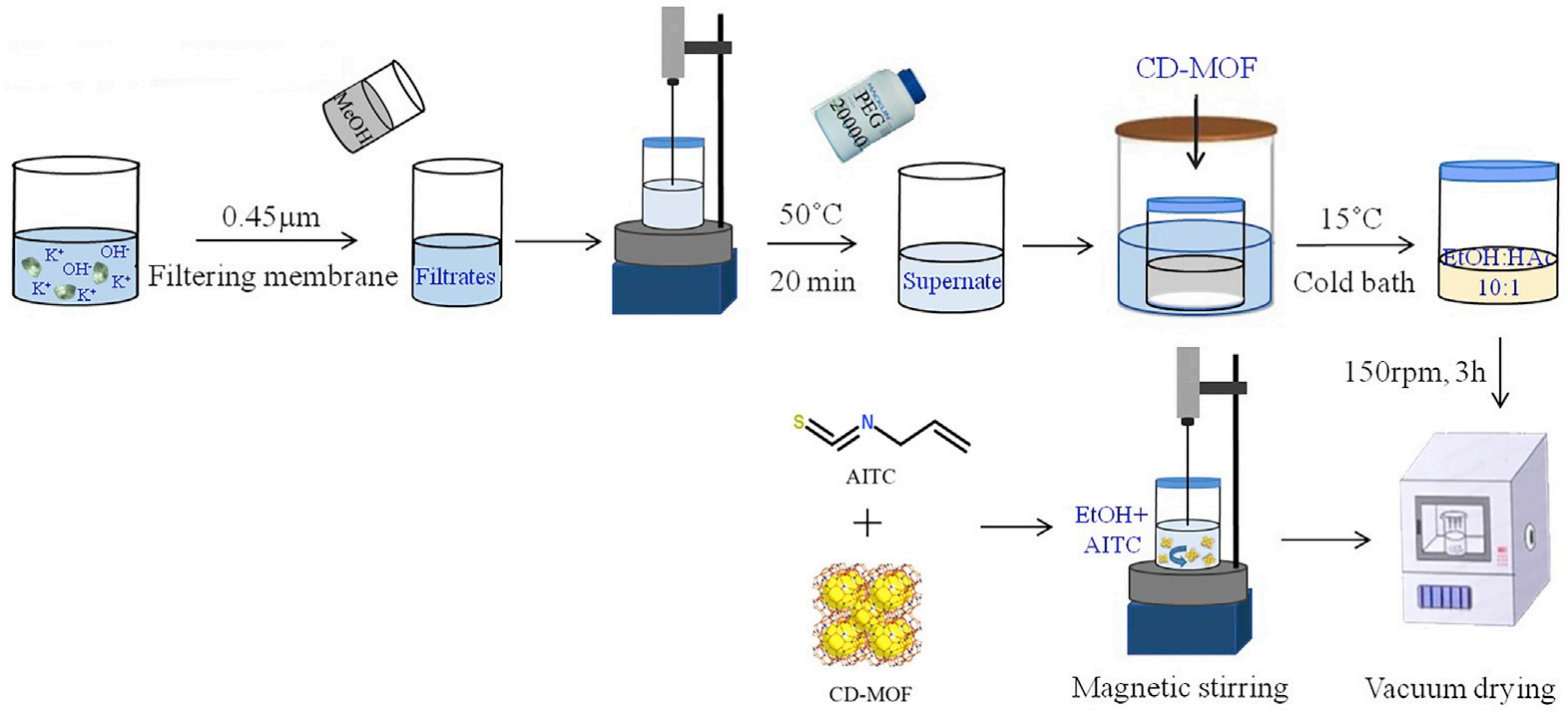
Schematic diagram of the preparation of CD-MOF-AITC

Allyl isothiocyanate (AITC), also known as mustard oil, is one of the most common natural isothiocyanates, existing mainly in brassicaceous vegetables (See Scheme 1). It has generated much enthusiasm among researchers to explore the application of AITC based on its beneficial biological and pharmacological activities. It had been found to have various important biological activities, including the potential anti-tumor activity, and the therapeutic effect for non-alcoholic fatty liver disease through activating sirtuin 1/adenosine monophosphate-activated protein kinase pathway and inhibiting the nuclear transcription factor-κB pathway to improve fatty liver and its inflammation.^1^^,^^2^^,^^3^^,^^4^ Meanwhile, AITC could reduce the level of blood glucose, total cholesterol, triglyceride and creatinine in type 2 diabetes rats induced by the high-fat diet or streptozotocin, and had anti-diabetic, antioxidant and anti-inflammatory activities.^5^ In our previous studies, it had been proven that AITC could treat chronic obstructive pulmonary disease through regulating multidrug resistance-associated protein 1, reacting oxide species and reducing glutathione levels.^6^^,^^7^ However, AITC had bad characteristics because of its strong irritation, volatility, easy to oxidize and deterioration in the air.^8^ Therefore, cyclodextrin, polyacrylate-coated kaolin embedding, and other technologies were used to improve the adverse characteristics of AITC, which was often added to food as an antibacterial agent and antioxidant, playing a role of antibacterial and anti-corrosion.^9^^,^^10^ Furthermore, it had been reported that the bioavailability of AITC in rats was high. It is mainly distributed in kidney, lung, liver, and other tissues. Its targeting to lung was low.^7^^,^^11^^,^^12^

Pulmonary drug delivery, which is one of the most effective ways to perform the effects of a drug or nutrient, can maximize the local lung drug dosage, because the structure of the alveoli provides a large surface area for the material exchange to the outside world, avoiding gastrointestinal degradation and liver first pass effect.^13^^,^^14^ However, the clearance function of phagocytes and mucocilia in the lungs to foreign bodies exposed in the airway is the biggest obstacle to drug delivery in the lungs.^15^ Therefore, in order to achieve effective atomization, maximize lung deposition, and reduce the clearance of macrophages, the complex treatment, and modification of particles are usually required.^16^ As an advanced pulmonary drug delivery agent, dry powder inhaler has the advantages of no-propellant, convenience, simple operation, low equipment cost, and good stability.^17^^,^^18^ The atomization performance of dry powder mainly depends on the aerodynamic diameter, surface morphology, and hygroscopicity of particles. Particle size is one of the most important factors, which affect the efficiency of drug deposition in lung. It is generally considered that the aerodynamic diameter (Da) of 1∼5 μm is ideal, because the drug is transported to the deeper part of lung by gravity deposition, and deposited on the surface of trachea, bronchus, and alveoli.^19^^,^^20^

Cyclodextrin metal-organic framework (CD-MOF) is considered a green, renewable framework material, linked by the coordination of the secondary face hydroxyl groups on alternating D-glucopyranosyl residues to one of the alkali metal cations to form body-centered cubic extended structures.^21^ In recent years, CD-MOF was mostly used as an oral drug delivery carrier. The stability, bioavailability, and half-life of ibuprofen, lansoprazole, azilsartan, and other drugs could be improved by loading into CD-MOF.^22^^,^^23^^,^^24^^,^^25^ The latest research demonstrated that budesonide could be loaded into CD-MOF to prepare dry powder inhalant, because the particle size of CD-MOF could be controlled within the range of 1∼5 μm of inhalable particle size, and the morphology of CD-MOF was highly uniform and regularly.^26^ Therefore, CD-MOF could be used as an ideal carrier for pulmonary drug delivery. Compared with other aerosols, the homogenous nanoscale pores of CD-MOF could effectively improve aerosolization performance. The biocompatibility of CD-MOF was good since cyclodextrin, the organic linker of CD-MOF was widely used as an excipient in pulmonary drug delivery, which was proven to be safe.^27^^,^^28^ The application of CD-MOF broadened the direction for the development of a new carrier for pulmonary drug delivery.

Cyclodextrins (CDs) are cyclic oligosaccharides mainly including β-, and γ-CD are composed of 7 and 8 glycosyl units, respectively, linked by 1, 4 bonds that comprise a family of cyclic oligosaccharides. Although β- and γ-CD-MOF both could be applied in the pharmaceutical industry, their structure and function were remarkably different. There were two unique cages in γ-CD-MOF molecular structure: (i) spherical voids (1.7 nm in diameter) shaped by six γ-CDs (simplified as (γ-CD)6) and (ii) cavities with a diameter of 0.8 nm formed by aspectant γ-CD pairs (simplified as (γ-CD)2).^29^ Generally, the poorly soluble drug molecules tended to enter the cavity in hydrophobic γ-CD pairs. If the drug loading solution was in high concentration, drug molecules were likely to form nano-clusters in the hydrophilic spherical voids. In addition, γ-CD-MOF could maintain its crystal features and morphologies after drug loading.^24^^,^^30^ β-CD-MOF could form a laminated structure, a “T” shape with a unique bowl-like pore. It had been investigated that fluorouracil and quercetin could be loaded into the bowl-like pore of β-CD-MOF, because β-CD-MOF contains the channel configuration, which could enclose drug molecules in β-CD-MOF.^31^ Most importantly, it had revealed that β-CD-MOF and γ-CD-MOF had different protective capacities, the stability of dimercaptosuccinic acid was greatly improved by β-CD-MOF, but decreased by γ-CD-MOF.^32^

In this study, AITC was prepared as dry powder inhalation by loading CD-MOF to enhance the pulmonary delivery to treat chronic obstructive pulmonary disease. β-CD-MOF and γ-CD-MOF were used and designed by optimizing the synthesis conditions and further engineering into inhalable particles as the carrier of AITC for lung delivery. The drug loading and characterization for those two kinds of CD-MOF-AITC were carried out to screen a more suitable dry powder inhalant carrier. Then, the quality of the dry powder inhalant was evaluated by measuring the particle size, *in vitro* lung deposition, the release behavior in simulated lung fluid, the distribution *in vivo* fluorescence imaging, and the biosafety. Finally, through the establishment of the human bronchial epithelial (16HBE) cell model under the normal condition and stimulating by cigarette smoke extract (CSE) condition, the permeability of dry powder inhalant *in vitro* was investigated, and the absorption in lung was evaluated.

## Results and discussion

### Influences of incubation time, temperature, and molar ratio on drug loading

**Figure 1:**
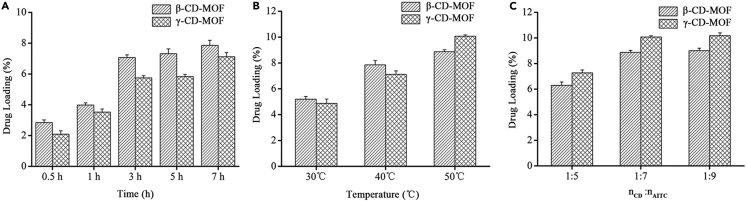
Influence of drug loading conditions on the drug loading of AITC (A-C) A: incubation times; B: temperatures; C: the molar ratio of CD-MOF and AITC (*n*_CD_ : *n*_AITC_). (Values are represented as mean ± SD. n = 3.)

The incubation time, temperature, and molar ratio were optimized to obtain the better drug loading efficiency of CD-MOF-AITC (Figure 1). The incubation time had a great influence on the drug loading in the two kinds of CD-MOF in Figure 1A. With time increasing, the drug loading boosted gradually. Moreover, the drug loading of AITC in β-CD-MOF was always higher than that in γ-CD-MOF at any point in time. The drug loading of AITC in β-CD-MOF reached the peak in 3 h, and there was no significant difference with the extension of time (p > 0.05). However, the drug loading of AITC in γ-CD-MOF reached the peak in 7 h that was in a range from three hours to seven hours. It indicated that the loading mechanism of AITC in those two kinds of CD-MOF might be different. Besides, the temperature factor had an impact on drug loading as well (Figure 1B). The drug loading increased with the improvement of the temperature, because the temperature enhanced the activity of AITC in the solution. When the temperature was increased from 40 °C to 50 °C, the drug loading of AITC in γ-CD-MOF was higher than that in β-CD-MOF. It might be due to the different structures between β-CD-MOF and γ-CD-MOF. Compared with β-CD-MOF, γ-CD-MOF had a larger cavity and external specific surface area, which made the drug loading greatly increased. There were two unique cages in γ-CD-MOF molecular structure: (i) spherical voids (1.7 nm in diameter) shaped by six γ-CDs and (ii) cavities with a diameter of 0.8 nm formed by aspectant γ-CD pairs. γ-CD-MOF with cavities (1.7 nm and 0.8nm in diameter) was prone to embedding drug molecules that were trapped and difficult to be washed off.^29^ Whereas the structure of β-CD-MOF was a layer that blocked the cavity and increased the difficulty of AITC to enter.^32^

The molar ratio had an influence on the dynamic balance between inside and outside of AITC in CD-MOF. As shown in Figure 1C, the drug loading of AITC was in balance in a ratio of 1: 7 (*n*_CD_: *n*_AITC_). According to the drug loading results, as the loading time and temperature increased, the loading amounts also enhanced. However, AITC was easy to be oxidized and degraded in a short time because it was a volatile oil. Considering the stability of the drug, the loading time and temperature were limited. It was concluded that the optimized drug loading of AITC in CD-MOF was achieved at the condition with 50 °C, 1:7 of molar ration (*n*_CD_: *n*_AITC_), and 7 h of reaction time. The drug loading of AITC was 9% and 10% in β-CD-MOF and γ-CD-MOF respectively.

### Characterization of allyl isothiocyanate, cyclodextrin metal-organic framework, and cyclodextrin metal-organic framework-allyl isothiocyanate

#### Morphology and particle size of CD-MOF and CD-MOF-AITC

**Figure 2:**
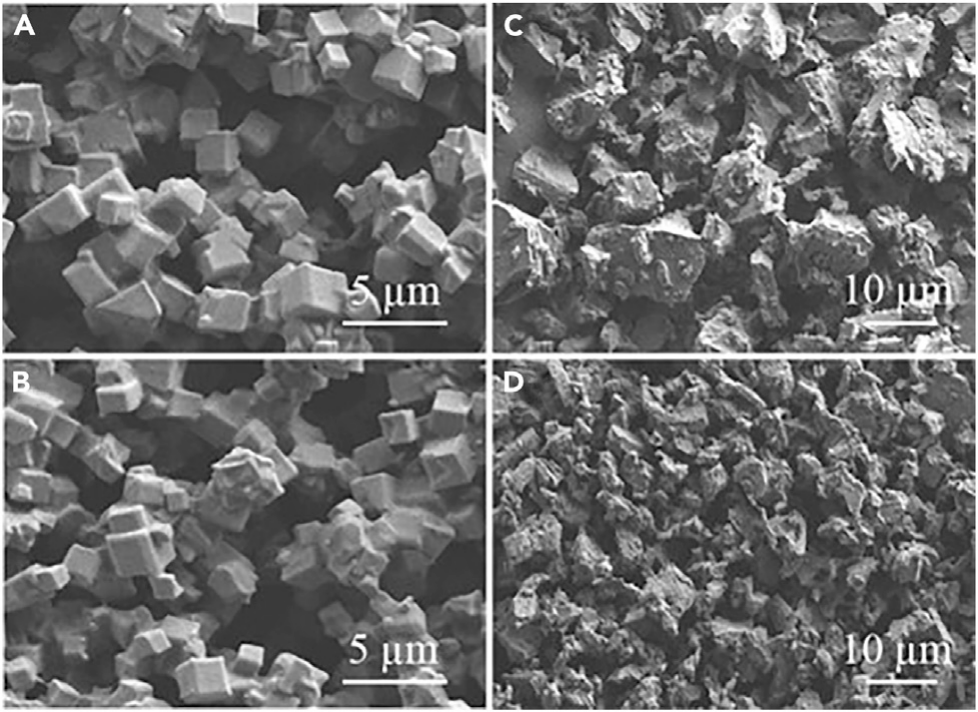
SEM morphologies of CD-MOF and CD-MOF-AITC (A-C) A: γ-CD-MOF; B: γ-CD-MOF-AITC; C: β-CD-MOF; D: β-CD-MOF-AITC.

Before and after AITC loading, the two kinds of CD-MOF powders were all white loose powders, with no obvious difference in appearance. However, the morphology was significantly different as shown in Figure 2. The crystals of γ-CD-MOF and γ-CD-MOF-AITC were both cubic, manifesting that the existence of AITC did not result in the morphology changes in γ-CD-MOF (Figures 2A and 2B). The particle sizes of γ-CD-MOF-AITC were in the range of 1∼5 μm, which met the requirements of dry powder inhalant. On the microscale, it showed a considerable inclusion capability. Generally, the poor soluble drug molecules tended to enter the cavity in hydrophobic γ-CD pairs. If the drug loading solution was in high concentration, drug molecules were likely to form nano-clusters in the hydrophilic spherical voids.^29^ On the contrary, the microstructure of β-CD-MOF changed greatly after drug loading in Figures 2C and 2D. The crystals of β-CD-MOF after AITC loading were smaller than before. Its structure was changed after the loading of AITC and stirring for a long time. It might be due to the laminated structure of β-CD-MOF. And there was no covalent bond with K^+^ in flank. After drug loading and a long time of magnetic stirring, the structure of the crystal might be not maintained and become smaller than before.^32^

#### X-ray diffraction analysis

**Figure 3:**
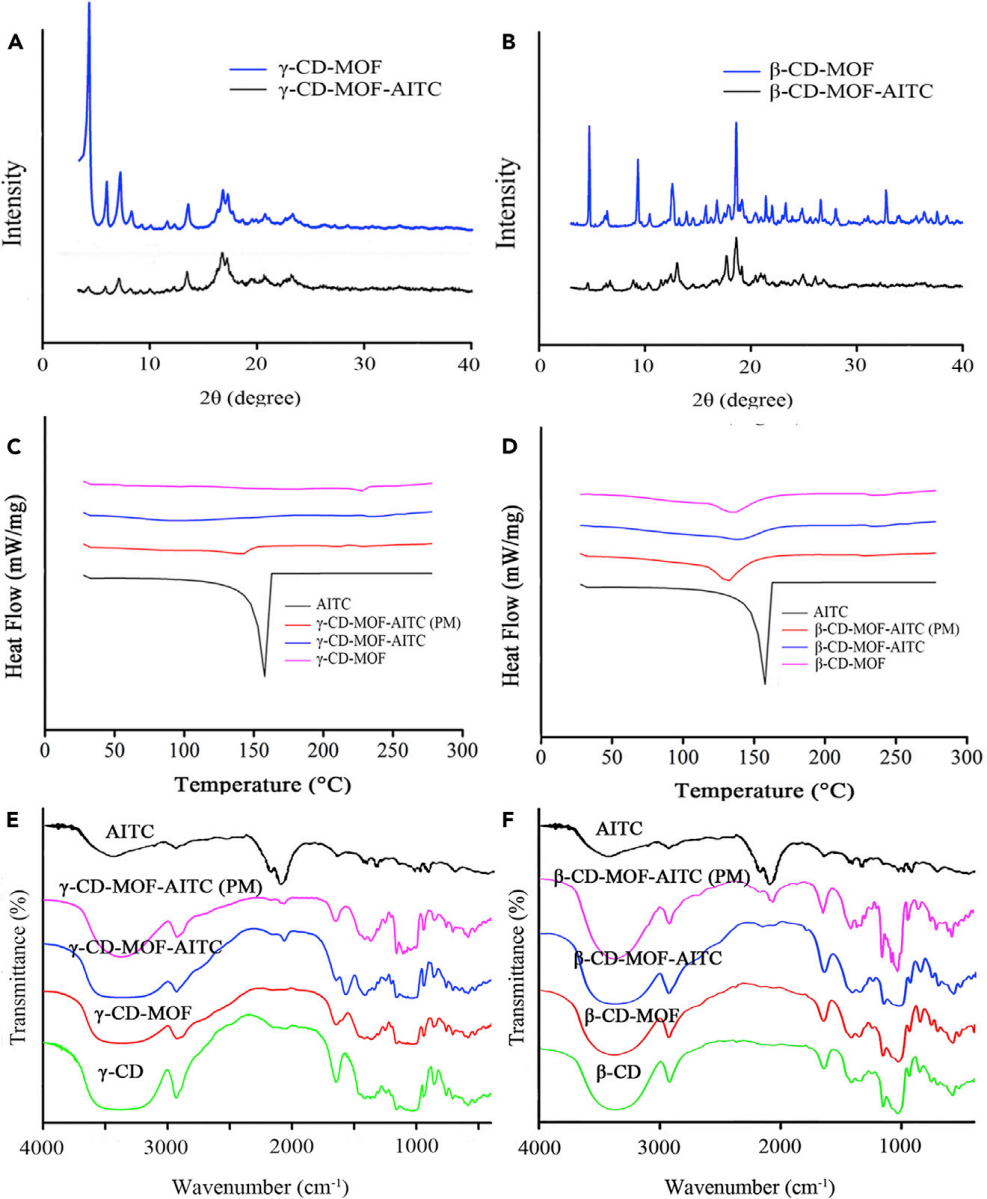
Characterization of AITC, CD-MOF, and CD-MOF-AITC (A-E) XRD pattern (A and B), DSC pattern (C and D), and FTIR spectra (E and F) of AITC, β-CD-MOF, β-CD-MOF-AITC, γ-CD-MOF, and γ-CD-MOF-AITC.

In order to determine whether the crystal structure of CD-MOF could be destroyed after loading AITC, the XRD characteristics of β-CD-MOF, β-CD-MOF-AITC, γ-CD-MOF, and γ-CD-MOF-AITC were performed respectively and the results were presented in Figures 3A and 3B. The inherent diffraction peaks of typical crystallization patterns of CD-MOF were shown. The intrinsic diffraction peaks of γ -CD-MOF were 4.0 °, 7.0 °, 13.0 °, and 16.7 °, which were consistent with the previous reports.^24^^,^^33^ After loading, the characteristic diffraction peaks of γ-CD-MOF were retained, and only the intensity of some characteristic peaks weakened obviously. It might be caused by slight leakage of γ-CD in ethanol during the process of AITC loading.^34^ The diffraction peaks of β-CD-MOF appeared at about 4.7 °, 9.4 °, 18.6 ° and 32.7 °.^35^ After loading into AITC, some characteristic diffraction peaks of β-CD-MOF weakened or disappeared. The crystallinity of β-CD-MOF changed greatly, which was consistent with the results as seen from the SEM images.

#### Differential scanning calorimetry analysis

The thermal behaviors of two kinds of CD-MOF, which before and after loading, were studied with DSC patterns. As shown in Figures 3C and 3D, the heat absorption peak of AITC occurred at 158 °C, but disappeared completely in the DSC curves of β-CD-MOF-AITC and γ-CD-MOF-AITC, which was similar to the previous report.^32^ It demonstrated that AITC distributed in the frameworks of β-CD-MOF and γ-CD-MOF. In contrast, there were small endothermic peaks in the physical mixtures between AITC and the two kinds of CD-MOF. The peaks shifted from 158 °C to 134 °C and 145 °C, respectively, suggesting that there was a weak mutual effect between the two components without generating a new phase.

#### Fourier transform infrared analysis

The interaction between AITC and CD-MOF could be discussed by observing the changes in bonding between functional groups through the FTIR spectrometer (Figures 3E and 3F). There was a wide absorption peak in a 3030∼3670 cm^−1^ region, which was the characteristic peak of CD-MOF, reflecting the stretching vibration of the -OH group in glucose unit.^36^ Compared with single CD, the peak at 2850∼3000 cm^−1^ (the stretching vibration of -CH_2_) in CD-MOF spectrum was obviously narrower, which might be related to the change of molecular conformation after the binding of metal ions K^+^ and CD. 1020∼1150 cm^−1^ was attributed to -C-O-C-stretching vibration, which might be due to the linking of 1, 4-glycosidic bond of CD in CD-MOF.^25^ In comparison to that of CD, the amplitude of such absorption peak in CD-MOF weakened. As a consequence, the absorption peaks of β-CD-MOF and γ-CD-MOF both existed and changed, and it suggested that those two kinds of CD-MOF were synthesized successfully and the skeleton structure of CD remained unchanged during the synthesis of CD-MOF. The main characteristic peaks of AITC were the stretching vibration of -N=C=S double bond at 2097 cm^−1^ and 2168 cm^−1^ and the stretching vibration of R-CH = CH_2_ at 1647 cm^−1^. Compared with AITC, the absorption peak almost disappeared at 2168 cm^−1^ in the two kinds of CD-MOF-AITC spectrum. And the absorption peak weakened significantly at 2097 cm^−1^ in the γ-CD-MOF-AITC spectrum and disappeared in the β-CD-MOF-AITC spectrum. It was assumed that AITC was covered entirely by the two kinds of CD-MOF while no characteristic peaks of AITC were detected. The stretching vibration of the carbon-oxygen single bond in CD-MOF-AITC moved from 1029 cm^−1^ to 1022 cm^−1^, and it might be due to the hydrogen bond and/or electrostatic attraction between AITC and γ-CD-MOF. Similarly, it was reported that the band at about 1030 cm^−1^ shifted to a shorter wavelength, suggesting the strengthening of the hydrogen bonding or electrostatic attraction.^24^^,^^32^

In summary, it showed that AITC was successfully loaded into β-CD-MOF and γ-CD-MOF from the characterization of the CD-MOF samples before and after AITC loading. The crystal morphology of γ-CD-MOF-AITC could be hold, while it changed greatly in β-CD-MOF-AITC in comparison to that of CD-MOF as shown in SEM and XRD images. Moreover, by comparing the drug loading of the two kinds of CD-MOF, γ-CD-MOF had more advantages in drug loading efficiency. γ-CD-MOF was used as a dry powder inhalant carrier for further research.

### *In vitro* deposition studies

**Figure 4:**
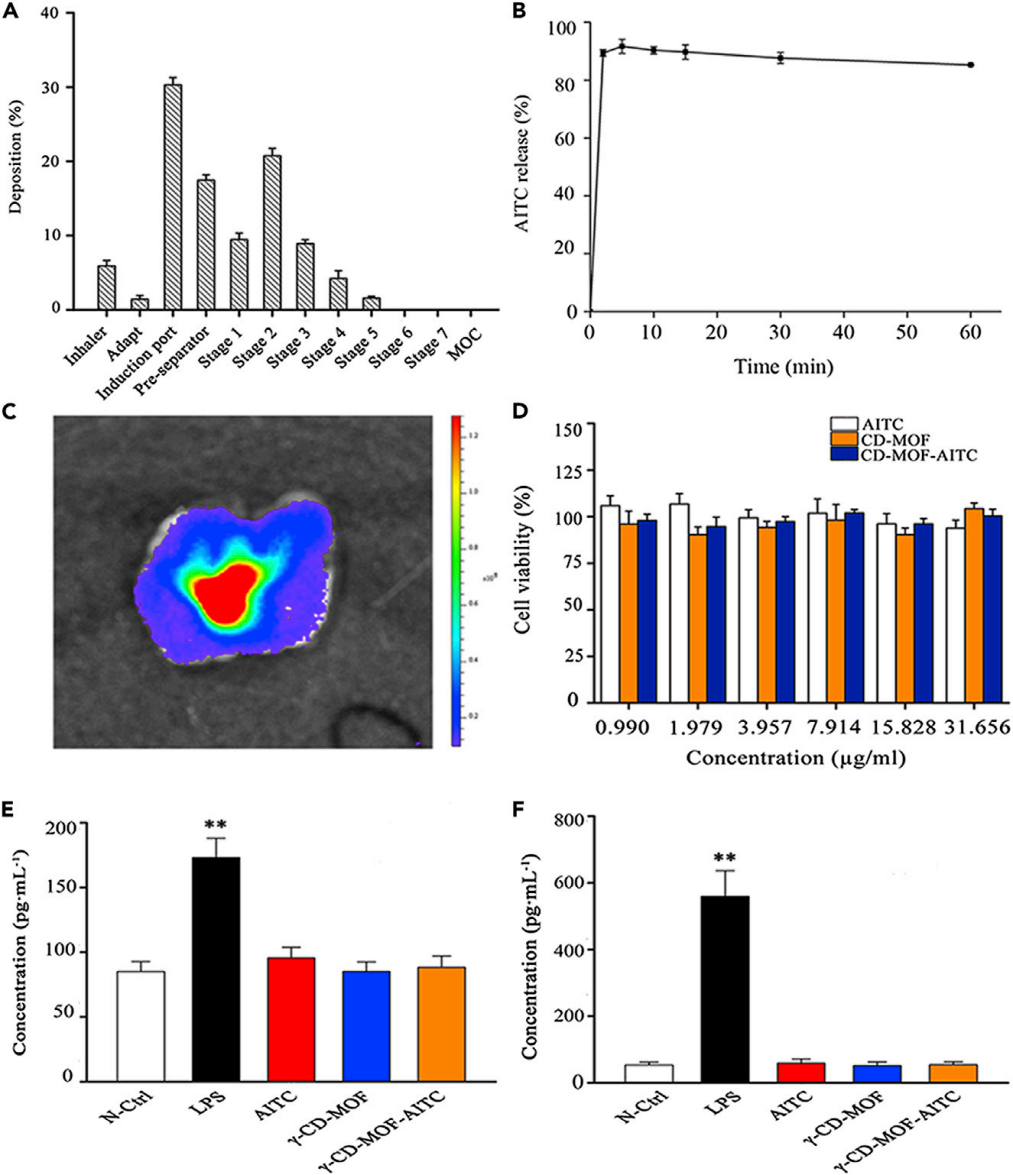
Quality and biological safety evaluation of dry powder inhalant (A-F) Percent of drug deposition (A, n = 3), the dissolution profiles (B, n = 3), lung deposition (C), the biological safety evaluation (D, n = 3), levels of pro-inflammatory cytokine IL-1β (E, n = 8) and TNF-α (F, n = 8) of γ-CD-MOF-AITC. (Values are represented as mean ± SD. ∗∗p < 0.01 vs. control group.)

Aerosolized dry powders were transported in the respiratory airways through dispersion, atomization, and lung deposition, and then absorbed by the lung tissue and blood vessels after inhalation to produce desired pharmacological effects. Thus, the deposition of the particles was the key factor for the AITC formulation to achieve efficient lung deposition. To investigate the dispersibility and aerodynamic properties of the γ-CD-MOF-AITC dry powders, the aerosol performance of the powders was characterized by the NGI. As shown in Figure 4A, less than 10% of the γ-CD-MOF-AITC sample remained in the capsules, it indicated that the particles were easily aerosolized and passed through the device. The MMAD, an important parameter that represents the aerodynamic properties of respirable particles, was calculated as 4.72 μm for γ-CD-MOF-AITC. GSD was used to describe the shape of the powder particle size distribution curve. It was generally believed that the closer such parameter value was to 1, the narrower the particle size distribution of the dry powder inhaler. As shown in Table 1, the GSD of the γ-CD-MOF-AITC particles was 1.31. Combined with the result of SEM, the particle sizes of γ-CD-MOF-AITC were in the range of 1∼5 μm and were concentrated distribution. The particles between 2 and 3 μm in size were around 60%. To sum up, the dry powder inhaler of γ-CD-MOF-AITC basically met the requirements of pulmonary inhalation, and it could be used as a pulmonary delivery carrier to deliver AITC to the lungs through the investigation of powder properties.

**Table 1:** The aerodynamic parameters for γ-CD-MOF-AITC dry powder inhaler (n = 3)

Parameters	γ-CD-MOF-AITC
Emitted fraction (EF, %)	68.60 ± 1.58
Fine particle fraction (FPF, %)	17.81 ± 0.69
MMAD (μm)	4.72 ± 0.06
GSD	1.31 ± 0.02

### *In vitro* release study

The release of γ-CD-MOF-AITC dry powder in the lung was investigated using simulated lung fluid as the release medium to simulate the internal environment of the lung. As shown in Figure 4B, the prepared γ-CD-MOF-AITC was a quick-release dry powder inhalant. AITC was completely released within 5 min with a release rate of about 90%. It was consistent with the result that γ-CD-MOF could be disintegrated rapidly in the aqueous solution.

### Fluorescence imaging *in vivo*

The crystal morphology of γ-CD-MOF did not change before and after loading from the result of SEM. Therefore, RhoB was used as a fluorescence agent, and γ-CD-MOF-Rhob was synthesized to replace γ-CD-MOF-AITC. The pulmonary distribution of dry powder inhaler particles was traced at Figure 4C. After 5 min of administration, a strong fluorescence signal could be observed in the lungs of mice. And it indicated that the particles were successfully lodged in the lung, and γ-CD-MOF was rapidly disintegrated to release RhoB. Due to the porous structure and suitable aerodynamic diameter of γ-CD-MOF from the result of SEM and the investigation of powder properties, it could be seen that the powder could be well distributed in the lung and reached into the deep.

### Biological safety evaluation

#### Cytotoxicity test

In order to study the biocompatibility and cytotoxicity of γ-CD-MOF-AITC dry powder inhalation, the effects of AITC, γ-CD-MOF, and γ-CD-MOF-AITC on the survival rate of 16HBE cells were determined by CCK-8 kit. As shown in Figure 4D, the cell growths were not significantly inhibited by AITC, γ-CD-MOF, and γ-CD-MOF-AITC in the concentration range of 0.990-31.656 μg/mL, and the cell survival rates were all above 90%. It indicated that γ-CD-MOF had good cytocompatibility. It could be used as a potential new dry powder inhalant carrier to safely deliver AITC to the lung.

#### The tolerance of lung

The biosafety of γ-CD-MOF-AITC dry powder inhalation was studied in healthy mice. The potential inflammatory response was investigated by quantifying the concentrations of IL-1β and TNF-α in BALFs. Compared with the negative control group, the concentrations of IL-1β and TNF-α in the LPS-administered group as a positive control group were significantly increased (p < 0.05). In comparison with the negative control group (p > 0.05), there was no significant difference in the concentrations of IL-1β and TNF-α detected in BALFs in the γ-CD-MOF and γ-CD-MOF-AITC groups. It showed that the dry powder inhalant with γ-CD-MOF as carriers had a good lung tolerance and no obvious stimulation to lung tissue after repeated and long-time administration.

### Permeability studies

**Figure 5:**
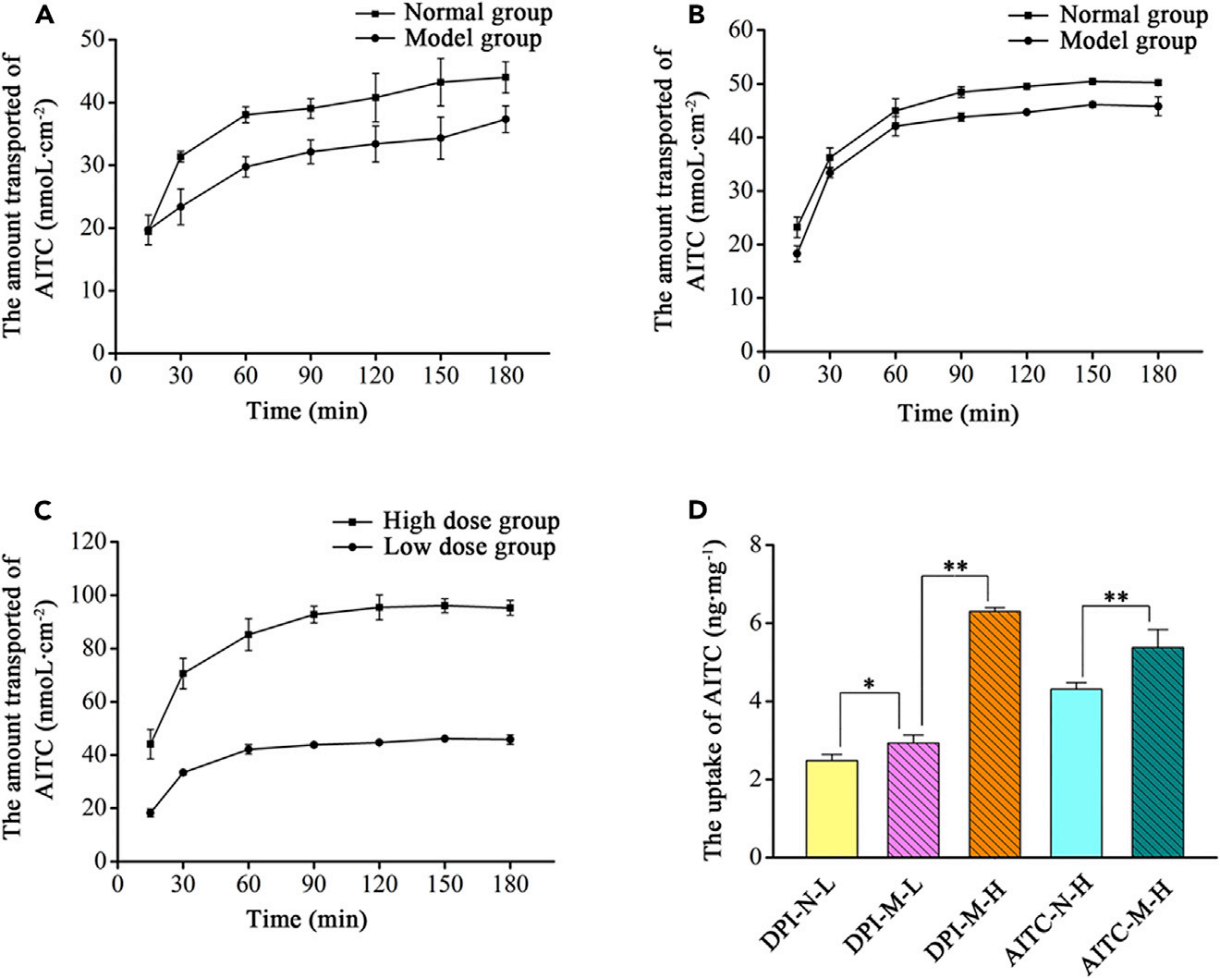
Permeability studies of AITC and CD-MOF-AITC (A-C) The cumulative transfer volume of AITC, γ-CD-MOF-AITC in the normal group and model group (A, B, C) and the uptake of AITC (D) in 16HBE epithelial cell layers. (A: AITC in the normal group and model group; B: γ-CD-MOF-AITC in the normal group and model group; C: Low- and high-dose γ-CD-MOF-AITC in model group; Values are represented as mean ± SD. ∗∗p < 0.01; ∗p < 0.05. n = 3.)

The amount of AITC from the top of the cell layer to the basal side was measured in a 16HBE cell model, and its absorption in the lung was assessed by the apparent permeability coefficient (Papp). As shown in Figure 5A, the transport of AITC in the cell model within 3 h was time-dependent. The cumulative transport volume of AITC in the normal group was higher than that in the model group at all time points, and the Papps of AITC in the normal group and the model group were 3.37 × 10^−6^ cm/s and 2.51 × 10^−6^ cm/s respectively. And they were both larger than 1 × 10^−6^ cm/s. It indicated that AITC was well absorbed in lung and could be used for pulmonary administration. The lung absorption of γ-CD-MOF-AITC dry powder inhalation was evaluated by establishing cell models of the normal group and model group, respectively (Figures 5B and 5C). It revealed that the accumulative transshipment volume of AITC in the γ-CD-MOF-AITC dry powder inhalation of the normal group was higher than that in the model group within 3 h, which was consistent with the result of AITC administration. However, the difference in accumulative transshipment volume reduced significantly. The transport of AITC in the cell model was time- and concentration-dependent, and the cumulative transport at each time point enhanced with the increase of the dose of dry powder inhalant. Meanwhile, the drug concentrations in the cells of the model group were significantly higher than those of the normal group after the administration of AITC and γ-CD-MOF-AITC dry powder inhalant as could be seen in Figure 5D. With the growth of the dose of dry powder inhalant, the drug concentration in the cell model increased significantly, and the drug intake in cell model in the preparation group was higher than that in the AITC group.

According to the above results, the cumulative transport volume of AITC in the model group was lower than that in the normal group, but the intake was higher than that in the normal group. Based on the preliminary research results of the research group, CSE stimulation could change the expression of the exocytocyte transporter multidrug resistance-associated protein 1 and other related proteins in 16HBE cells.^24^^,^^32^ This might result in differences in the transport and uptake of AITC between the normal group and the model group, and it could be predicted that the absorption of AITC in the lung tissue of normal and chronic obstructive pulmonary disease might be different. The cell model of the model group was more representative of the follow-up investigation. By comparing the transport and uptake results of AITC and γ-CD-MOF-AITC dry powder inhalers in the model group, the effect of the loading of AITC into γ-CD-MOF on the drug absorption in the lung was investigated. According to the above results, the drug penetration rate that loaded into γ-CD-MOF was faster, the cumulative transport volume was boosted significantly, and the drug intake was also higher than that of AITC administration in the cell model. Dry powder inhalation was better absorbed in the lung, and γ-CD-MOF may promote the absorption of AITC in the lung.

### Conclusions

In summary, CD-MOF was used as an AITC carrier for pulmonary delivery to enhance pulmonary delivery for treating chronic obstructive pulmonary disease. In this study, β-CD-MOF and γ-CD-MOF could both be used to carry AITC with the optimal loading conditions (50˚C, *n*_CD_: *n*_AITC_ = 1:7, 7 h). And γ-CD-MOF was more suitable as a dry powder inhalant carrier of AITC based on the content of drug loading and the characterization. The particle size of the γ-CD-MOF-AITC dry powder inhaler was between 1 and 5 μm, and the MMAD was 4.72 μm, which accorded with the aerodynamic characteristics of lung inhalation. AITC could be released quickly and completely in simulated lung fluid, with a good rapid release effect. After the administration of γ-CD-MOF-RhoB powder, a strong fluorescence signal could be observed in the lungs of mice, and powder particles could effectively deposit to the deep lung. γ-CD-MOF-AITC dry powder inhaler had no significant toxicity to 16HBE cells, good local lung tolerance, and no stimulation to lung tissue. Meanwhile, γ-CD-MOF could promote the absorption of AITC, and the permeability of AITC in the form of the dry powder inhaler was better than only AITC. These results indicated that γ-CD-MOF could be used as a dry powder inhaler carrier to safely deliver AITC to lung and increase its pulmonary absorption.

### Limitations of the study

In this study, CD-MOF was used as an AITC carrier for pulmonary delivery to enhance pulmonary delivery for treating chronic obstructive pulmonary disease. Orthogonal experiment should be used to optimize the drug loading of AITC in CD-MOF. Experiments with the modification of CD-MOF are needed to improve the flowability of powders and increase the rate of drug deposition in the lungs. Meanwhile, the pharmacodynamic evaluation of CD-MOF-AITC dry powder inhalation is needed to be further studied.

## STAR★Methods

### Key resources table

**Table:** 

REAGENT or RESOURCE	SOURCE	IDENTIFIER
**Chemicals, peptides, and recombinant proteins**		

Allyl isothiocyanate (AITC)	Anhui Haibei import and export Co., Ltd (Anhui, China)	CAS: 57-06-7
β-CD	Shanghai Mackin Biochemical Co., Ltd (Shanghai, China)	CAS: 7585-39-9
γ-CD	Shanghai Mackin Biochemical Co., Ltd (Shanghai, China)	CAS: 17465-86-0
Rhodamine B	Alfa Aesar	CAS: 81-88-9

**Experimental models: Cell lines**		

Human bronchial epithelial (16HBE) cell	Fuxiang Biological Technology Company (Shanghai, China)	CB455254989

**Experimental models: Organisms/strains**		

Male Kunming mice	Biomedical Research Institute of Nanjing University	SCXK2017-001

### Resource availability

#### Lead contact

Further information and requests for resources and reagents should be directed to and will be fulfilled by the lead contact, Dianlei Wang ((Tel:/Fax: 18956057159; Email: dlwang@ahtcm.edu.cn (D. Wang)).

#### Materials availability

Raw materials used in the study were commercially available. Anything they need to reproduce the work is in the method details.

### Experimental model and subject details

The male Kunming mice, weighing 20 ± 2g and aging 8 weeks old, were purchased from Biomedical Research Institute of Nanjing University. All mice were randomly assigned to five groups and kept under a standard 12-hour light/ dark cycle with *ad libitum* food and water. The animal protocol was approved by the animal ethics committee of Anhui University of Chinese Medicine. 16HBE cells were purchased from Fuxiang Biological Technology Company (Shanghai, China). The cells were cultured in DMEM supplemented with 10% fetal bovine serum, and then incubated at 37 °C in an incubator with 5% CO_2_.

### Method details

#### Materials

β-CD, γ-CD and PEG 2000 were purchase from Shanghai Mackin Biochemical Co., Ltd (Shanghai, China). Allyl isothiocyanate (AITC, >98%) was provided by Anhui Haibei import and export Co., Ltd (Anhui, China). All chemicals and regents were of analytical grade or higher.

#### Preparation of CD-MOF-AITC

The synthetic procedure for β-CD-MOF referred to method in a reported article.^32^ In an optimal procedure of micrometer sized CD-MOF synthesis, β-CD (4.54 g, 4 mmol) and potassium hydroxide (1.80 g, 32 mmol/L) was dissolved in purified water (46 mL). The solution was filtered through polyvinylidene fluoride organic filter membrane (0.45 μm) and then mixed with the same volume methanol in 50 °C for 20min. Then the supernatant was transferred, and PEG 20000 (12 mol/mL) was added as a particle regulator in 15 °C for overnight incubation in an airtight glass reactor. The precipitates were harvested after centrifugal separation and then adjusted pH to neutral. After washing with ethanol and CH_2_Cl_2_ twice, the obtained crystalline precipitation was air-dried at 40 °C for 12 h.

The synthetic protocol for γ-CD-MOF was adapted from a previously reported procedure.^30^ For details, γ-CD (2.59 g, 2 mmol/L) and potassium hydroxide (a molar ratio of 1:8) were both firstly dissolved in purified water and filtered through polyvinylidene fluoride organic filter membrane (0.45 μm). Secondly, methanol was added into the above solution in 50 °C for 20 min. Then the supernatant was transferred, and PEG 20000 (12 mol/mL) was added in 15 °C for overnight incubation in an airtight glass reactor. The precipitates were harvested, adjusting pH to neutral and washing with ethanol and CH_2_Cl_2_ twice. Finally, the obtained crystalline precipitation was air-dried at 40 °C for 12 h.

AITC-loaded CD-MOF (CD-MOF-AITC) was prepared by magnetic stirring heating method. β-CD-MOF or γ-CD-MOF (200mg), and AITC (a molar ratio of 1:7) were added in 5 mL ethanol solution in a magnetic stirring heater for drug loading in 40 °C for 0.5, 1, 3, 5, and 7 h, respectively. The crystals were harvested after centrifugal separation. After washing with ethanol thrice, the obtained crystalline precipitation was vacuum drying at 40 °C for 12 h (The detail was shown in Scheme 1). Different incubation temperature (30 °C, 40 °C, 50 °C) and molar ratio (1:5, 1:7, and 1:9) were used successively to load AITC in β-CD-MOF or γ-CD-MOF on the basis of the best drug-loading condition.

#### Determination of drug loading

CD-MOF-AITC was dissolved with ultrapure water to release AITC and then determined by UV spectrophotometer (Specord 600, JENA, Germany) at 248 nm wavelength. The content of drug loading was the ratio between the weight of AITC and the weight of CD-MOF-AITC. All measurements were carried out in triplicate.

#### Characterization of CD-MOF and CD-MOF-AITC

The scanning electron microscopy (SEM, 75000F, JEOL, Japan) was employed to observe the surface morphology of the powders of CD-MOF and CD-MOF-AITC. The particle size and distribution of the samples were analyzed and calculated from the images using Image Pro Plus software. Prior to imaging, samples were fixed onto a metal plate using conductive adhesive tape and coated with gold for 60 s. The X-ray diffraction patterns (XRD) of β-CD-MOF, β-CD-MOF-AITC, γ-CD-MOF, and γ-CD-MOF-AITC were analyzed using an X-ray diffractometer (D2 PHASER, Bruker AXS, Inc., Germany) to characterize the crystal structure. Samples were irradiated with monochromatic CuKα radiation and analyzed over a 2θ angle range of 3–40 °. PXRD was collected with the tube voltage of 40 kV, tube current of 40 mA and a scan speed of 0.1 s per step. The differential scanning calorimetry (DSC) of CD-MOF before and after loading AITC and the physical state of the physical mixture of AITC and CD-MOF were characterized. An amount of 10mg of each sample was weighed in steel pans, and then heated from 25 to 150 °C at a heating rate of 10 °C/min. The fourier transform infrared spectrometer (FTIR) was measured using a Nicolet IS10 spectrometer (Thermo Nicolet, Inc., Waltham, MA, U.S.A.). The sample was fully mixed with potassium bromide and pressed into tablets. Each sample was scanned in the wavenumber of 400–4000 cm^−1^.

#### Quality evaluation of γ-CD-MOF-AITC

*In vitro* pulmonary deposition performance of γ-CD-MOF-AITC was determined using a Next Generation Impactor (NGI 170, Copley Scientific, England). About 10 mg of γ-CD-MOF-AITC powders were accurately weighed and filled into size 3 gelatin capsules. Before measurement, each impactor stage was covered with 1% (v/v) dimethicone in nhexane to trap the particles. The air flow rate was 60 L/min. This operation was repeated ten times. The effective aerodynamic cutoff diameter for each impactor stage at this flow condition was calibrated according to the operation instructions: Stage 1, 8.06 μm; Stage 2, 4.46 μm; Stage 3, 2.82 μm; Stage 4, 1.66 μm; Stage 5, 0.94 μm; Stage 6, 0.55 μm and Stage 7, 0.34 μm. The particles deposited on the adaptor, induction port, preseparator and all impactor stages were recovered and dissolved in the mobile phase. The concentration of AITC in the solutions was determined by HPLC equipped with Agilent HC-C18 column (4.6 × 150 mm, 5 μm) at 35 °C with the flow rate of 1.0 mL/min and the mobile phase composed of methyl alcohol and water (60:40, v/v) with the injection volume of 10 μL. The detection wavelength was 248 nm. The doses of the powder on the adaptor, induction port, pre-separator and all impactor stages were represented as emitted dose (ED). The amounts of powders filled into the capsules were total dose (TD). Fine particle dose (FPD) was the dose deposited lower than 4.46 μm on impactor stages 2–7. The emitted fraction (EF) and fine particle fraction (FPF) were calculated by the following equations: EF (%) = ED/ TD ×100%; FPF (%) = FPD/ED ×100%. The mass median aerodynamic diameter (MMAD) and the geometric standard deviation (GSD) of the samples were calculated using the software (Coply CITDAS V3.10).

##### *In vitro* release

The *in vitro* release of AITC from γ-CD-MOF-AITC was determined. In brief, 25 mg of γ-CD-MOF-AITC was redispersed in 10 mL of simulated lung fluid (pH 7.4, 1L containing 0.2g/L MgCl_2_⋅6H_2_O, 6.0 g/L NaCl, 0.3g/L KCl, 0.32 g/L Na_2_HPO_4_⋅12H_2_O, 0.28 g/L CaCl_2_, 0.57 g/L CH_3_COONa, 2.6 g/L NaHCO_3_, 0.1 g/L C_6_H_5_Na_3_O_7_⋅2H_2_O, 0.02% Tween 80) at 37 °C with a constant gentle stirring. At pre-determined time points (0-1h), the amount of AITC released was determined.

##### Fluorescence imaging *in vivo*

The pulmonary distribution of dry powder inhaler particles of γ-CD-MOF-AITC was traced by Rhodamine B (RhoB) as a fluorescence agent. γ-CD-MOF-RhoB was synthetized by magnetic stirring heating method. Before the experiment, mice were fasted for 12 h and anesthetized by intraperitoneal injection of 1% pentobarbital sodium (40 mg/kg). After superficial anesthesia, the mice were fixed on the operating table with medical tape, and the pulmonary delivery device DP-4 was used to inject γ-CD-MOF-RhoB into the trachea and deliver it to the lung. Five minute later, the complete lung tissue was removed, rinsed quickly in normal saline and dried with filter paper. The fluorescence imaging of lung tissue was recorded by IVIS Spectrum small animal optical imaging system (Perkinelmer, America). The excitation wavelength and emission wavelength of RhoB were 550 nm and 580 nm respectively.

#### Biological safety evalution

##### Cytotoxicity test

16HBE cells were purchased from Fuxiang Biological Technology Company (Shanghai, China). The cells were cultured in DMEM supplemented with 10% fetal bovine serum, and then incubated at 37 °C in an incubator with 5% CO_2_. The experimental groups were added with different concentrations of AITC, γ-CD-MOF, and γ-CD-MOF-AITC solution respectively, and the control group was added with serum-free DMEM. After 24 h of incubation, 10 μL CCK-8 solution was added, and the culture was continued for 4 h. OD value of each sample at 450 nm was determined. The cytotoxicity of γ-CD-MOF was investigated by calculating the cell survival rate of each group.

##### The tolerance of lung

Male mice were purchased from Biomedical Research Institute of Nanjing University. All mice were kept under a standard 12-hour light/ dark cycle with *ad libitum* food and water. The animal protocol was approved by the animal ethics committee of Anhui University of Chinese Medicine. Forty male mice were randomly assigned to five groups. The experimental groups were added with lipopolysaccharide (LPS), AITC, γ-CD-MOF, and γ-CD-MOF-AITC solution, and the control group was untreated. AITC (20mg/kg), γ-CD-MOF (200mg/kg), and γ-CD-MOF-AITC (200mg/kg) powders were actuated into the lungs by insufflation using a dry insufflator device (DP-4R, Penn-century, USA). The mice were anesthetized by intraperitoneal injection of 1% pentobarbital sodium (40 mg/kg) for light anesthesia before administration, 3 times a week for 3 consecutive weeks. And 24 h after the last administration, the mouse was euthanized through injecting intraperitoneally with 1% pentobarbital sodium (90 mg/kg). The right lung was ligation, and the precooled PBS was injected into the main bronchus The bronchoalveolar lavage fluids (BALFs) were collected and the concentrations of IL-1β and TNF-α in BALFs were determined by ELISA.

#### Permeability studies

16HBE cells in model group were treated with 5% CSE for 24 h. Administration groups were pretreated with AITC (480 μM), γ-CD-MOF-AITC (4mg and 8mg) in normal and model group. The uptake of AITC in 16HBE epithelial cell layers was investigated through measuring the concentrations of AITC and protein. And the results were expressed as (NG drug)/ (Mg protein).

### Quantification and statistical analysis

All experiments were conducted at least thrice, and the mean values and standard deviations were determined. The experimental data were analyzed using analysis of variance (ANOVA) and were expressed as mean values ± standard deviations. Differences were considered at a significance level of 95% (p < 0.05) and 99% (p < 0.01). Pearson’s correlation coefficients among the parameters were calculated using the Statistical Package for the Social Sciences (SPSS) version 17.0 software.

## Data Availability

All the data needed to evaluate the conclusions of this work are detailed in the main text. The article did not generate any software or code. Any additional information required to reanalyze the data reported in this paper is available from the lead contact upon request.
